# Molecular Basis of Hyperammonemic Encephalopathy in Fibrolamellar Hepatocellular Carcinoma

**DOI:** 10.7759/cureus.33750

**Published:** 2023-01-13

**Authors:** Rodrigo Cañada T Surjan, Thais M de Lima, Heraldo P de Souza, Marcel Cerqueira C Machado, José C Ardengh

**Affiliations:** 1 Surgery, Hospital 9 de Julho, São Paulo, BRA; 2 Clinical Emergencies Laboratory LIM 51, University of São Paulo, São Paulo, BRA; 3 Surgery and Anatomy, Hospital das Clinicas - Faculdade de Medicina de Ribeirão Preto, Ribeirão Preto, BRA; 4 Imaging Diagnostic, Universidade Federal de São Paulo, São Paulo, BRA; 5 Digestive Endoscopy Service, Hospital Moriah, São Paulo, BRA

**Keywords:** physiopathology, hyperammonemia-encephalopathy, encephalopathy, metabolic encephalopathy, fibrolamellar hepatocellular carcinoma

## Abstract

Hyperammonemic encephalopathy is a potentially fatal condition associated with fibrolamellar hepatocellular carcinoma. The mechanism involved in hyperammonemia in patients with fibrolamellar carcinoma was unclear until a possible physiopathological pathway was recently proposed. An ornithine transcarboxylase dysfunction was suggested as a result of increased ornithine decarboxylase activity induced by c-Myc overexpression. This c-Myc overexpression resulted from Aurora kinase A overexpression derived from the activity of a chimeric kinase that is the final transcript of a deletion in chromosome 19, common to all fibrolamellar carcinomas. We performed the analysis of the expression of all enzymes involved and tested for the mutation in chromosome 19 in fresh frozen samples of fibrolamellar hepatocellular carcinoma, non-tumor liver, and hepatic adenomatosis. The specific DNAJB-PRKACA fusion protein that results from the recurrent mutation on chromosome 19 common to all fibrolamellar carcinoma was detected only in the fibrolamellar carcinoma sample. Fibrolamellar carcinoma and adenomyomatosis samples presented increased expression of Aurora kinase A, c-MYC, and ornithine decarboxylase when compared to normal liver, while ornithine transcarbamylase was decreased. The proposed physiopathological pathway is correct and that overexpression of c-Myc may also be responsible for hyperammonemia in patients with other types of rapidly growing hepatomas. This gives further evidence to apply new and adequate treatment to this severe complication.

## Introduction

Ammonia is a constituent of body fluids generated in the intestine by bacterial hydrolysis of nitrogen compounds, muscular amino acid transamination, purine nucleotide cycle, and metabolic processes mainly in the kidneys and liver. Ammonia and bicarbonate are condensed in the hepatic mitochondria to produce carbamoyl phosphate to initiate the urea cycle, the most important mechanism of blood ammonium removal. When the blood level of ammonia increases, it enters the central nervous system (CNS) excessively and becomes toxic to the brain [1].

Astroglial cells are the only CNS cells that metabolize ammonia. Ammonia is condensed with glutamate to form glutamine. As the level of glutamine increases, it results in astrocyte swelling, cerebral edema, and intracranial hypertension. When astrocytes are continuously exposed to ammonia, they may undergo phenotypic transformation into Alzheimer´s type II astrocytes with reduced proliferative activity [2]. Moreover, elevated concentrations of ammonia in CNS promote oxidative stress. Glutamine and ammonia exposure to astrocytes increases reactive oxygen species production, another possible cause of astrocyte swelling responsible for neurotoxicity [3].

Liver failure is the cause of 90% of hyperammonemia cases in adults. Those not related to liver failure may be divided into two groups: cases with increased ammonia production and cases with decreased elimination [1].

Increased ammonia production may occur in progressive multiple myeloma and infections by urea-producing bacteria. Rare causes include starvation, total parenteral nutrition, gastrointestinal bleeding, and seizures. Reduced elimination occurs mainly in metabolic disorders like urea-cycle disorders, pyruvate metabolism errors, organic acidurias, impaired fatty acid oxidation, dibasic aminoaciduria, and congenital portosystemic shunt [4].

Since 2009, reports have demonstrated the association of fibrolamellar hepatocellular carcinoma (FLHCC) with hyperammonemia. Nevertheless, none of those articles reached a definite explanation for hyperammonemia in FLHCC patients. Berger et al. theorized that portosystemic shunts were accountable [5]. Alsina et al. suggested that intrahepatic shunting and lack of clearance of nitrogenous compounds by tumor cells were responsible [6].

In 2017, Surjan et al. proposed a new physiopathological pathway to hyperammonemia in patients with FLHCC [7]. According to the theory, all FLHCC present a single and recurrent heterozygous deletion in chromosome 19 that results in a chimeric protein DNAJB1-PRKACA (a catalytic subunit of protein kinase A) [8]. The DNAJB1-PRKACA kinase is probably both necessary and sufficient for the carcinogenesis of FLHCC and results in Aurora kinase A (AURKA) overexpression within the tumor, as previously demonstrated [9].

Elevated levels of AURKA upregulate c-Myc transcription, affecting cellular proliferation and ATP production, important factors in FLHCC tumorigenesis [10]. c-Myc overexpression leads to increased ornithine decarboxylase (ODC) activity. This results in increased ornithine consumption to polyamines synthesis, reducing ornithine bioavailability that results in urea cycle disorder due to ornithine transcarboxylase (OTC) dysfunction and consequent hyperammonemia [10].

The proposal of this physiopathological pathway to HE in an FLHCC patient allowed innovative treatment with complete neurocognitive recovery. However, the described process was not proved by analysis of involved enzyme activities and RNA expression.

In this study, fresh frozen tissue samples of non-tumor liver parenchyma, FLHCC, and one hepatic adenomatosis in a patient that developed HE without liver dysfunction were submitted to analysis of expression of ODC, c-Myc, OTC, and AURKA and tested for the chromosome 19 deletion.

This article was previously posted to the Research Square preprint server on 07 Jun 2021; DOI: 10.21203/rs.3.rs-482483/v1; PPR353709

## Case presentation

Three fresh frozen tissue samples were used: an FLHCC in a 33-year-old male patient with multiple hepatic tumors and diffuse peritoneal carcinomatosis that developed severe and refractory hyperammonemic encephalopathy (ammonia blood level: 312 mcml/L (reference interval: 9-30 mcmol/L)), a non-tumor non-cirrhotic liver (ammonia blood level: 26 mcmol/L) and a rapid growing hepatic adenomyomatosis in a 44-year-old female patient that developed hyperammonemia (ammonia blood level: 26 mcmol/L) submitted to percutaneous ultrasonography-guided diagnostic hepatic lesion biopsy. All tissue samples were obtained by Quick-Core needle biopsies (Cook Group Inc. Bloomington, Indiana). Samples were immediately submitted to cryopreservation with liquid nitrogen.

Reagents

TRizol (Invitrogen. Carlsbad, California. USA), diethylpyrocarbonate (DEPC), 2x SYBR Green Reaction Mix (Invitrogen), Super Script III RT/Platinum Taq Mix (Invitrogen), ROX Reference Dye (Invitrogen), glyceraldehyde 3-phosphate dehydrogenase (GAPDH), β-2 microglobulin (B2M), ethylenediaminetetraacetic acid (EDTA), and ethidium bromide solution (EtBr).

Real-time PCR (RT-PCR)

RT-PCR was used to determine messenger RNA (mRNA) levels of proteins of interest. Total RNA was extracted from 100 mg of frozen liver with TRIzol reagent following the manufacturer’s instructions. RNA was dissolved in DEPC-treated water and quantified spectrophotometrically at 260 nm. One hundred nanograms of total RNA was used for each real-time PCR reaction, which was performed in StepOne equipment (Applied Biosystems Inc. Foster City, California). RT-PCR was performed in a 15 µl reaction mixture containing 7.5 µl 2x SYBR Green Reaction Mix, 0.3 µl each primer, 0.3µl Super Script III RT/Platinum Taq Mix (10 pmol/µl), 0.15µl ROX Reference Dye, and 5µl sample in water. cDNA synthesis was performed at 50ºC for 15 minutes followed by 35 cycles at 95ºC for 15 seconds, annealing temperature for 30 seconds, and 72ºC for 30 seconds. Quantification was performed by the 2-DDCT method using GAPDH and B2M as housekeeping genes [10,11]. The primer sequences and annealing temperature are listed in Table 1. Primers were designed using GeneRunner Software (Hastings Software Inc. Hastings, NY). The DNAJB1-PRKACA fusion transcript primer sequence was obtained by Graham et al. (Table 1) [8].

**Table 1:** Sequences and annealing temperature of primers used for real-time PCR PCR: polymerase chain reaction; AURKA: Aurora kinase A; ODC ornithine decarboxylase; OTC: ornithine transcarboxylase

Gene	Sense primer (5’ – 3’)	Reverse primer (5’ – 3’)	Annealing temperature	Fragment size (bp)
DNAJB1/ PRKACA	GGAGAAGTTCAA GGAGATCGCT	CAAGTGGGCTG TGTTCTGAG	65	163
AURKA	CTCAGCGGGTCT TGTGTCCTTC	TTGATGCCAGTT CCTCCTCAGG	58	219
c-Myc	CCACAGCAAACC TCCTCACAGC	ACTTGACCCTCT TGGCAGCAGG	58	122
ODC	GAAAGTTGCCAG AGCACATCCC	GGTACAGCCGC TTCCTACATGG	56	190
OTC	CTCCAGGCTTTC CAAGGTTACC	TCTGTCAGCAG GGACACCATG	58	200
GAPDH	TGCCAAATATGA TGACATCAAGAA	GGAGTGGGTGT CGCTGTTG	60	121
B2M	GATGAGTATGCC TGCCGTGTG	CAATCCAAATGC GGCATCT	60	114

Agarose gel electrophoresis

PCR amplicons fragments were separated via 1% agarose gel electrophoresis. The gels were prepared by dissolving the agarose in the TBE buffer (100 mM Tris, 100 mM boric acid, 2 mM EDTA). To visualize the DNA, 1.5 µl of (10 mg/ml) EtBr was added to the 100 ml 1% agarose solution. To load the samples, the DNA was mixed in equal volume ratios with the agarose gel loading buffer. Electrophoresis was performed at 100 V. The DNA was detected using UV light and the size of the DNA was determined using the standard 100bp DNA ladder.

Compliance with ethical standards

Tissue samples used in this research were obtained from already available biological material and patients signed an informed consent form before the use of the samples. Ethics clearance was obtained from the Ethics Research Committee at the University of São Paulo Medical Faculty and all experiments were performed in accordance with relevant guidelines and regulations.

Results

**Figure 1:**
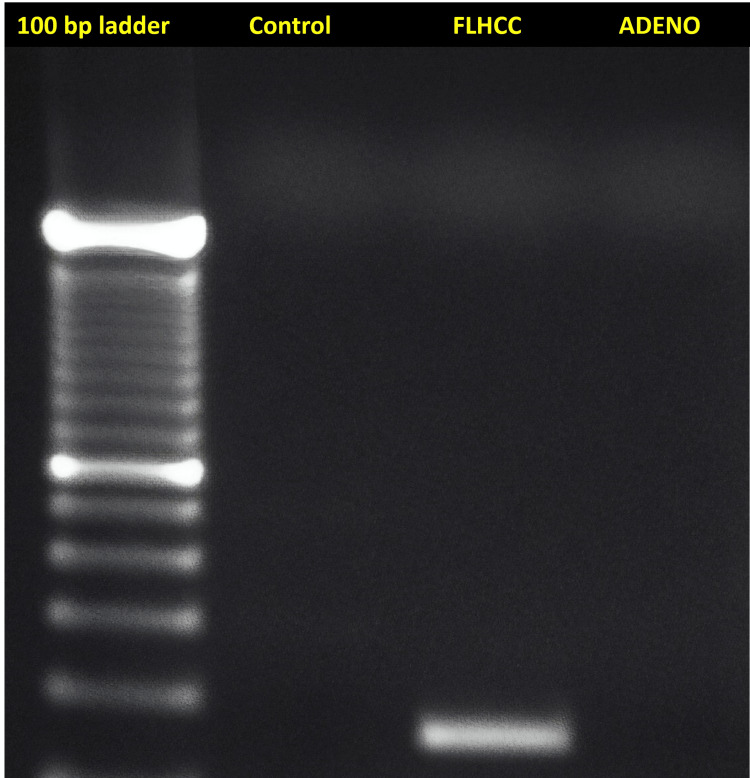
Representative gel image showing RT-PCR for DNAJB1-PRKACA fusion transcripts; the DNAJB1-PRKACA fusion transcript is detected only in the FLHCC sample RT-PCR: real-time polymerase chain reaction; FLHCC: fibrolamellar hepatocellular carcinoma; adeno: adenomyomatosis

The DNAJB1-PRKACA fusion protein and mutation on chromosome 19 were detected only in the FLHCC sample (Figure 1).

**Figure 2:**
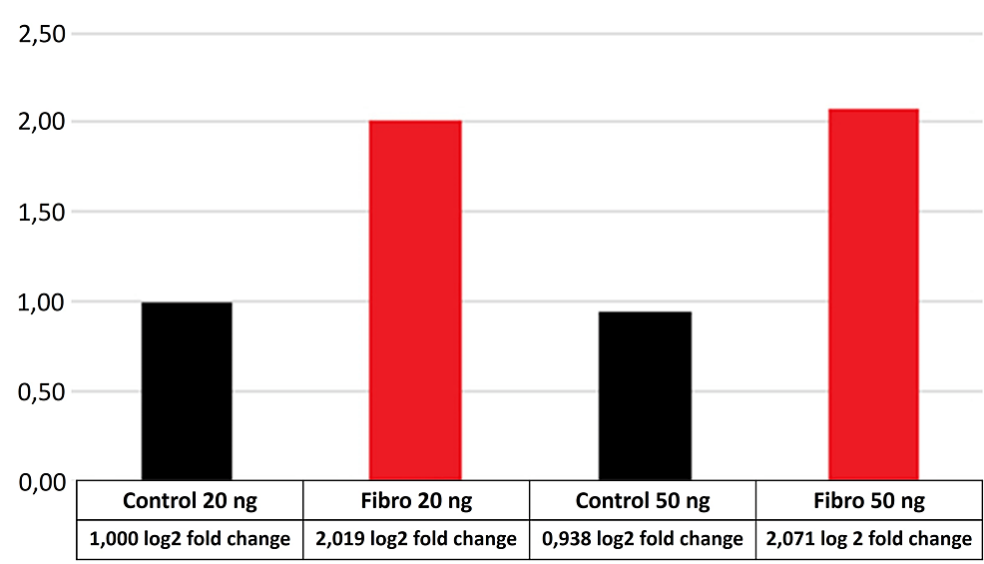
ODC expression on fibrolamellar carcinoma and normal liver samples using 20 and 50 ng of RNA in RT-PCR reactions Control: normal liver; Fibro: fibrolamellar carcinoma; OCD: ornithine decarboxylase; RT-PCR: real-time polymerase chain reaction

**Figure 3:**
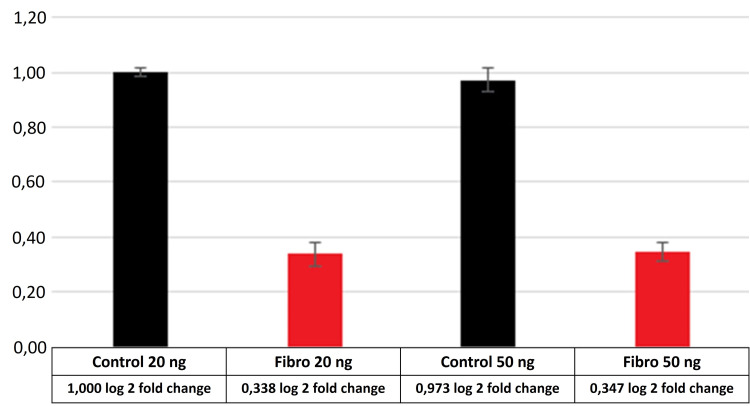
OTC expression on fibrolamellar carcinoma and normal liver samples using 20 and 50 ng of RNA in RT-PCR reactions Control: normal liver; Fibro: fibrolamellar carcinoma; OTC: ornithine transcarbamylase; RT-PCR: real-time polymerase chain

The relative expression versus Glyceraldehyde-3-Phosphate Dehydrogenase (GAPDH) of AURKA in FLHCC and adenomyomatosis samples presented increased expression of AURKA, c-Myc, and ornithine decarboxylase (Figure 2) when compared to normal liver. On the other hand, ornithine transcarbamylase was decreased in FLHCC and adenomyomatosis when compared to a non-tumor liver (Figure 3). There was no difference between FLHCC and adenomyomatosis in the expression of AURKA and c-Myc.

Samples analyses were performed by RT-PCR in triplicates. The results are expressed as relative expressions compared to GAPDH using the 2-DDCT method.

## Discussion

HE is a severe condition that must be suspected in patients that present progressive neurocognitive disorders, seizures, and coma even in the absence of liver failure. Rapid-onset, non-cirrhotic HE in adults can lead to significant brain injury and sequelae, and in most cases, proves to be fatal [3,4].

There are many different etiologies for non-hepatic HE, ranging from infection with urea-producing bacteria to late-onset urea cycle disorders or chemotherapy induction [4]. The association between HE and hepatic tumors dates to 1972 when Weber described a urea cycle disorder mimicking an ornithine carbamoyltransferase deficiency [12]. Different types of liver malignancies in non-cirrhotic patients (usually large and rapidly growing tumors) have also been accompanied by hyperammonemia [13,14]. However, FLHCC, despite being a rare liver malignancy, is often associated with hyperammonemia and encephalopathy [7].

The precise mechanism involved in HE in patients with FLHCC and other hepatic tumors has not been clearly identified in the literature until 2017 when Surjan et al. proposed a new physiopathological pathway to HE theorized when treating an FLHCC patient that developed severe hyperammonemia and coma that showed no signs of liver failure or portosystemic shunting and that did not present any inborn metabolism error detected in multi-gene panel genetic testing [7,15].

According to the theory, hyperammonemia would be the result of a urea cycle disorder due to a reduction of the activity of OTC because of ornithine consumption by polyamine synthesis, especially putrescine, spermine, and spermidine, caused by overexpression of ODC [7]. The reduction in the activity of OTC results in lower consumption of aspartate and carbamoyl phosphate that could be spared for the biosynthesis of nucleic acids [16]. Moreover, polyamines are ubiquitous small basic molecules that exert important gene regulation functions being important substrates for DNA stabilization and repair, essential to cellular growth [17]. Those are paramount to carcinogenic steps and represent crucial biological advantages in a malignant microenvironment [11,18].

A proportional overexpression of ODC parallel to OTC activity reduction had been previously described in hepatomas in rats [12]. Nevertheless, the reason for this increased expression of ODC had not been understood before.

The proposed explanation would be that, as all FLHCC present a single and recurrent deletion on chromosome 19 that results in a chimeric DNAJB1-PRKACA kinase that augments AURKA expression and, as previously demonstrated AURKA overexpression upregulates c-MYC (oncogene on chromosome 8q24 of cellular origin), the result would be increased ODC expression secondary to c-Myc signaling [8,10].

This proposed physiopathological pathway would explain why FLHCC patients often develop hyperammonemia and severe encephalopathy. To prove this theory, we performed RT-PCR to determine messenger RNA (mRNA) levels of proteins of AURKA, c-Myc, ODC, and OTC in a non-tumor non-cirrhotic liver sample and FHLCC sample. Both samples were submitted to DNAJB1-PRKACA fusion protein detection, which was only present in FLHCC. The results corroborated what the theory suggested.

Moreover, as previous reports demonstrated that non-FLHCC rapidly growing liver tumors could also result in HE due to ODC overexpression for polyamine synthesis and consequent OTC expression reduction, we performed the same analysis on a sample of rapidly growing adenomyomatosis in a patient that developed hyperammonemia and neurocognitive abnormalities. We found a similar gene expression of all proteins.

## Conclusions

In conclusion, these findings are paramount to better understanding the development of hyperammonemia and its potentially fatal complication, HE, in patients not only with FLHCC but probably other types of hepatic tumors. Recognizing a different physiopathological pathway than the more common hepatic encephalopathy to this subset of patients allows distinct and probably life-saving treatment options.
